# The use of virtual reality in the treatment of autism

**DOI:** 10.1192/j.eurpsy.2021.938

**Published:** 2021-08-13

**Authors:** G. Pontes, C. Varanda

**Affiliations:** Human Sciences, Universidade Paulista, Santos, Brazil

**Keywords:** virtual reality, Treatment, autism

## Abstract

**Introduction:**

The characteristics of the Autistic Spectrum Disorder involve deficits in social communication and repetitive patterns of behavior and that there is a growing interest in the use of new technologies for neurorehabilitation.

**Objectives:**

This research aimed to verify the possibility of using Virtual reality for the treatment of Autism.

**Methods:**

Scientific publications were selected from the PUBMED, ScieLO, LILACS and Google Scholar databases, written in Portuguese and English, with free access, between 2014 and 2019.

**Results:**

A total of 19 publications were identified. Concerning their design, 26,3% of them were experimental, 21,1% qualitative, 21,1% one-group pretest-posttest, 15,8% quasi-experimental, 10,5% descriptive and 5,2% of them were exploratory research studies. The studies focus on anxiety and phobias reduction, as well as teaching strategies to deal with stessful events. Eleven of the studies focused on the enhancement of independende and sef-confidence of the subjects. In five ot the studies the virtual reality was used with other technologies. In two of them the EEG was used along with virtual reality for detecting the cerebral region in activity during action.

**Conclusions:**

Virtual reality was defended in most of the studies because it can provide a safe environment and offering high control of variables. Although the results indicate the use of virtual reality for the reduction of anxiety and the promotion of self-confidence and independence which aims the social deficits in autism, more research is needed to investigate the use or impact of VR on repetitive behavior.

